# Passivated Porous Silicon Membranes and Their Application to Optical Biosensing

**DOI:** 10.3390/mi13010010

**Published:** 2021-12-22

**Authors:** Clara Whyte Ferreira, Roselien Vercauteren, Laurent A. Francis

**Affiliations:** Institute of Information and Communication Technologies, Electronics, and Applied Mathematics, UCLouvain, 1348 Louvain-la-Neuve, Belgium; clara.whyte@student.uclouvain.be (C.W.F.); laurent.francis@uclouvain.be (L.A.F.)

**Keywords:** porous silicon, membranes, atomic layer deposition, passivation, biosensor, optical sensing

## Abstract

A robust fabrication method for stable mesoporous silicon membranes using standard microfabrication techniques is presented. The porous silicon membranes were passivated through the atomic layer deposition of different metal oxides, namely aluminium oxide Al_2_O_3_, hafnium oxide HfO_2_ and titanium oxide TiO_2_. The fabricated membranes were characterized in terms of morphology, optical properties and chemical properties. Stability tests and optical probing noise level determination were also performed. Preliminary results using an Al_2_O_3_ passivated membranes for a biosensing application are also presented for selective optical detection of *Bacillus cereus* bacterial lysate. The biosensor was able to detect the bacterial lysate, with an initial bacteria concentration of 10^6^ colony forming units per mL (CFU/mL), in less than 10 min.

## 1. Introduction

Porous silicon (PSi) is a promising material for many applications, the most popular being the fields of diagnostics, therapeutics and photonics [1,2,3,4]. PSi is obtained through nanostructuration of bulk silicon substrate via electrochemical etching [5]. This process allows to obtain a high surface area (up to 800 m^2^/g) [6] and a versatile surface that can be chemically modified and tuned for different applications. Furthermore, PSi exhibits optical properties (i.e., photoluminescence and reflectance) that are highly sensitive to the presence of chemical and biological species, allowing its use for optical biosensing applications [7].

Membranes can be obtained from PSi by detaching the porous layer from the underlying bulk silicon substrate. The formation of this permeable barrier helps to overcome the infiltration limitations of close-ended PSi and extends its application to different fields, such as flow-through optical detection, drug delivery, microfluidics, energy conversion and electronics [8].

However, one of the issues with freshly-etched porous silicon is the presence of hydride terminated groups which makes the structure highly reactive and unstable [4,9,10]. In aqueous buffer the surface of the porous matrix is oxidized and subsequently dissolved. This is particularly challenging for application in aqueous media because the dissolution of the matrix leads to instability over time and reduction of the ultimate sensitivity of the devices [11].

To tackle this issue, different passivation techniques have been proposed aiming the minimization of the oxidation reactions. The most common approach to stabilize porous silicon is the thermal oxidation of the surface under controlled conditions [12]. Oxidized porous silicon can be further modified using conventional silanol chemistries [11], the so-called silanization [13]. In this case, Si-O-Si bonds are formed between the silanol groups on the surface and hydrolysed organosilane molecules of 3-aminopropyltriethoxysilane (APTES) or 3-aminopropyldimethylethoxysilane (APDMES) [14,15], obtaining better results in terms of passivation than purely oxidized surfaces [16]. Another strategy is binding carbon directly to silicon to obtain a highly stable surface. Carbonized PSi can be generated by reacting the porous surface with gas phase acetylene, obtaining the so-called “hydrocarbonized” porous silicon. These types of treated surfaces were found to have an increased stability even in an aqueous KOH environment [17]. However it renders the surface hydrophobic [11] and uses a highly flammable gas [17].

Atomic layer deposition (ALD) can also be used to passivate the large internal surface of porous silicon layers. This method is controlled by a surface-limited reaction: one atomic layer is deposited in a single cycle of reactant gases [18]. This technique holds several advantages such as a highly conformal coating (ideal for high-aspect ratio nanostructures such as deep porous silicon layers), precise thickness control down to the Ångström level via its self-limiting mechanism, and it allows deposition of a vast array of materials [19,20]. Previous studies have shown that ALD on a PSi layer can render the porous silicon surface more chemically stable [20].

This work proposes an ALD passivation method that can be applied to porous silicon membranes resulting in robust membranes, able to resist a flow-through operation. Three metal oxides are tested, namely HfO_2_, TiO_2_ and Al_2_O_3_. Their performance is compared to PSi membranes oxidized at 200 °C. The temperature of the oxidation was chosen as to minimize the deformation of the porous matrix. We demonstrate the uniform coating of metal oxides inside the PSi nanostructure using Energy Dispersive X-ray Spectroscopy (EDX) and X-ray photoelectron spectroscopy (XPS). The stability of the passivated membranes overtime in aqueous media is also analysed. We illustrate a possible application of the coated membranes with the optical detection of *Bacillus cereus* lysate, using the same protocol as previously described [21]. Other possible applications include filtering [22,23] and energy conversion [24].

## 2. Materials and Methods

### 2.1. Fabrication and Characterization of Porous Silicon Membranes

#### 2.1.1. Materials

Double-side polished, 3-inches diameter boron-doped silicon wafers (⟨100⟩, 0.8–0.9 Mω·cm, 380–400·μm) were acquired from Sil’tronix Silicon Technologies (Archamps, France). Aqueous hydrofluoric acid (HF, 49%) was obtained from Chem-Lab, NV (Zeldegem, Belgium), and absolute ethanol was purchased from VWR Chemicals (Leuven, Belgium). Phosphate buffered saline (PBS, 0.01 M phosphate, pH 7.4) was purchased from Sigma-Aldrich (St. Louis, MO, USA). The atomic layer deposition precursors, trimethylaluminium (TMA), tetrakis(dimethylamido)hafnium (TDMAHf) and tetrakis(dimethylamido)titanium (TDMAT) for Al_2_O_3_, HfO_2_ and TiO_2_ respectively, were purchased from SAFC Hitech (Bromborough, UK).

#### 2.1.2. Fabrication and ALD Passivation

**Figure 1:**
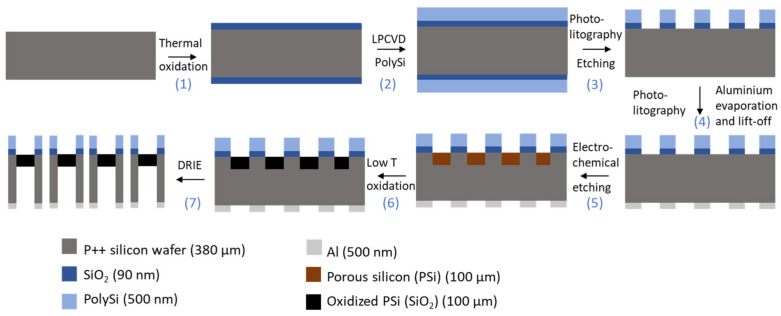
Schematic illustration of the manufacturing process of PSi membranes. Starting from a cleaned 3″-diameter highly doped silicon wafer, the process goes through the following steps: (**1**) thermal oxidation, (**2**) deposition of a polysilicon layer (PolySi) using Low Pressure Chemical Vapor Deposition (LPCVD); (**3**) positive photolithography on the frontside and opening of the PolySi and SiO_2_ layers using Reactive Ion Etching (RIE) and diluted HF respectively; (**4**) negative photolithography on the backside, evaporation of an aluminium mask and lift off of the resist; (**5**) formation of the porous silicon layer by electrochemical etching; (**6**) low temperature thermal oxidation; and finally (**7**) opening of the membrane using Deep Reactive Ion Etching (DRIE).

**Figure 2:**
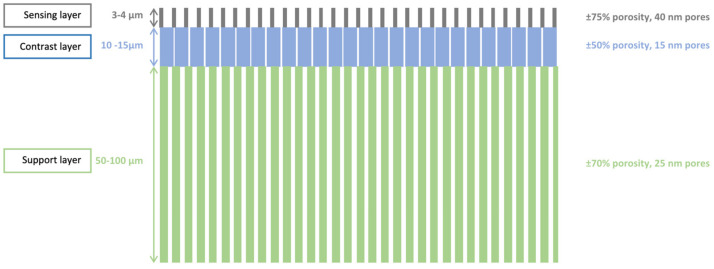
Schematic of a tri-layered PSi membrane, successively etched at 225, 50 and 100 mA/cm^2^.

Figure 1 illustrates the process flow used for the manufacturing of PSi membranes. To start, the silicon wafers were dipped in a freshly prepared piranha solution (H_2_O_2_:H_2_SO_4_, 2:5), followed by two immersions in continuously flowing deionized (DI) water during 20 min. Next, a 90 nm-layer SiO_2_ was grown using wet thermal oxidation at 1000 °C and a 500 nm-layer of polycrystalline silicon (PolySi) was deposited at 620 °C using Low Pressure Chemical Vapor Deposition (LPCVD). An i-line optical lithography with positive resist (AZ^®^ MiT™ 701, MicroChemicals GmbH, Ulm, Germany) provided masking for a Reactive Ion Ething (RIE) step, which was used to open the PolySi layer and remove it from the rear face. The SiO_2_ at the bottom of these patterns was removed using diluted HF (5% *v*/*v*). The patterned double-layer of SiO_2_ and Poly Si then acts as a mask during the electrochemical etching of the silicon [11]. A negative resist (AZ^®^ nLOF™ 5510, MicroChemicals GmbH, Ulm, Germany) was coated and patterned on the backside of the wafer, forming a lift off layer for the evaporation of aluminium. The metallization step took place in an e-gun VST and resulted in a 300 nm thick aluminium layer. This layer has a double purpose: it serves as a mask during the backside etching, and enables a better contact during the porosification step. This porosification was performed by anodization in a custom-built Teflon^®^ single bath etch cell, with a platinum coil as counter electrode, an aluminium working electrode and a potentiostat/galvanostat (PGSTAT302N from Metrohm, Antwerp, Belgium) as current source. The etching was carried out in HF:ethanol (3:1, in volume) electrolyte and consists in a four step process: (i) a sacrificial layer was etched at 200 mA/cm^2^ at 30 s and removed using a 1 M solution of KOH until no more reaction is visible; (ii) then a first layer, forming the sensing layer, was etched at 225 mA/cm^2^ for 50 s; (iii) a second layer, serving as contrast layer, was etched at 50 mA/cm2 for 880 s; (iv) and finally, a third thick layer was etched at 100 mA/cm^2^ for 3200 s, to ensure a mechanically stable membrane. After the anodization step, the silicon wafer was oxidized in a Heraeus heating oven under 1.5 L/min O_2_ and 1.2 L/min N_2_ flow at 200 °C for 30 min. The thus-formed thin oxide layer protects the porous silicon during the last step of the fabrication process, namely the Deep Reactive Ion Etching (DRIE) of the backside of the wafer. This DRIE etching is carried out until the porous silicon was visible from the backside. A schematic illustration of the resulting structure can be observed in Figure 2.

The fabricated membranes have two shapes: either 4 mm^2^ squares, or 3.14 mm^2^ circle. Each membrane is situated on a 1 cm^2^ die, and a 3-in. wafer contains 32 dies. The total thickness of the membrane ranged from 65 to 120 µm, depending on the membrane area, position on the wafer and different batches. Thinner membranes were avoided as they were easily mechanically deformed or cracked during the further processing steps.

Once fabricated, the membranes were divided in four batches: the first batch remained as is, while the three other batches were passivated using Atomic Layer Deposition (ALD) of three different metal oxides, namely HfO_2_, Al_2_O_3_ and TiO_2_. The oxide growths were carried out in a Fiji F200 ALD reactor (Veeco/CNT, Plainview, NY, USA) in exposure mode, meaning that the deposition chamber is isolated from the pump during the precursor pulses to allow a deep penetration of the precursors and a homogeneous coating throughout the porous matrix. All depositions were performed in thermal mode, at 100 °C for the HfO_2_ and Al_2_O_3_ and 150 °C for TiO_2_. These lower temperatures were selected to reduce thermal stresses and avoid the cracking and/or deformation of the PSi membranes. The first precursors are TMA, TDMAHf and TDMAT for Al_2_O_3_, HfO_2_ and TiO_2_ respectively. The second precursor is water vapor for all three types of deposition. The ALD parameters for the three metal oxides are detailed in Table 1. The numbers of cycles for each type of ALD was adjusted as to obtain layers with similar thicknesses, namely ~3 nm as measured per ellipsometry on flat silicon substrates submitted to the same deposition process.

#### 2.1.3. Optical Determination of Thickness and Porosity

The thickness and the porosity of the top layer of the PSi membranes was characterized using spectroscopic liquid infiltration method (SLIM) [11]. In short, this technique consists in measuring the reflective spectra of the PSi membrane both in air and in ethanol. It was carried out using a fibre-coupled Ocean Optics JAZ spectrometer and a 10-mW halogen light source. The optical spectra, characterized by Fabry-Pérot fringes, were analysed using a Fourier Transform over the 400 to 800 nm wavelength range. The position of peak returned by this analysis translated the effective optical thickness (EOT) of the top layer of the PSi membranes, which is equal to 2 *nL* (*n* being the refractive index of the layer and *L* its thickness). As the refractive indices of air, ethanol and silicon are known, a two-component Bruggeman effective medium approximation can fit the open porosity and the layer thickness based on the measured EOT values.

#### 2.1.4. Scanning Electron Microscopy (SEM) and Energy Dispersive X-ray Spectroscopy (EDX)

Scanning electron microscopy (SEM), using a Carl Zeiss Ultra 55 SEM, was used to observe the top layer and cross-section of the PSi membranes. The cross section allowed the measurements of each layer’s thickness, while the top views enabled the approximation of the average pore size, before and after passivation, using an image analyser routine on MATLAB (MathWorks) previously developed in the research group [25].

The SEM was also coupled with an Energy Dispersive X-ray Spectroscopy (EDX) detector, combined with the Esprit Software. These tools were used to analyse cross-sectional views of the membranes, and characterize conformity of the oxide deposition throughout the porous matrix depth.

#### 2.1.5. X-ray Photoelectron Spectroscopy (XPS)

X-ray Photoelectron Spectroscopy (XPS) analyses were performed to determine the chemical composition of the ALD films deposited on the pore surface. Therefore, XPS measurements were carried out on the top and back sides of the PSi membranes with an SSX 100/206 photoelectron spectrometer from Surface Science Instruments equipped with a monochromatized micro focused Al X-ray source (powered at 20 mA and 10 kV). The chamber pressure was fixed at 10^−6^ Pa and the data was collected at a 55° angle. The spot size was approximately 300 µm and the pass energy was set at 150 eV. In these conditions, the full width measured at half maximum (FWHM) of the Au 4f7/2 peak for a clean gold standard sample was about 1.6 eV. A flood gun set at 8 eV and a Ni grid placed 3 mm above the sample surface were used for charge stabilisation. The C-(C,H) component of the C1s peak has been fixed to 284.8 eV to set the binding energy scale. Data treatment was performed with CasaXPS program (Casa Software Ltd., Teignmouth, UK), some spectra were decomposed with the least squares fitting routine provided by the software with a Gaussian/Lorentzian (85/15) product function and after subtraction of a non-linear baseline. Molar fractions were calculated using peak areas normalised based on acquisition parameters and sensitivity factors provided by the manufacturer.

### 2.2. Optical Stability Measurements

The stability measurements were carried out in a custom-made polycarbonate fluidic cell. The cell was connected to a Fluigent LINEUP™ fluidic set up, which injected PBS at flow speed varying between 0.5 to 1 µL/min. Reflectivity spectra were recorded with the same equipment as detailed in Section 2.1.3. The spectra were acquired every 10 s for 4 h, with and integration time of 2000 ms and an averaging of five scans to reduce the noise level. The first hour of data was discarded as it is considered a transitional period during which the buffer penetrates the membrane. The remaining data was then analysed using Reflective Interferometric Fourier Transform Spectroscopy (RIFTS) [11]. Like the SLIM method, a Fourier Transform was applied to the optical spectra in order to quantify the EOT. The first six scans were averaged to obtain EOT_0_, the initial optical thickness of the sample. The evolution of EOT over time (EOT_t_) is expressed as a percentage change, as defined by the following equation

$$\Delta \mathrm{EOT}=\frac{{\mathrm{EOT}}_{t}-{\mathrm{EOT}}_{0}}{{\mathrm{EOT}}_{0}}\times 100\;\left[\%\right].$$

#### 2.2.1. Noise, Sensitivity and Limit of Detection Determination

Following the RIFTS data processing, the noise of each measurement was analysed using the following procedure (also described in Figure S1): First the data was fitted using a moving average filter (lowpass filter with filter coefficients equal to the reciprocal of the span, span = 0.15); then the fit was subtracted from the actual data; finally, the mean and standard deviation was computed for each sample based on these difference values. By averaging the standard deviation over all samples, the noise level σ_N_ can be determined.

The sensitivity S = ΔEOT/Δn [%/RIU] was also computed: First the EOT of PSi membranes was measured in different media (namely air, methanol, ethanol and isopropanol); the EOT was then plotted with respect to the refractive index of each media and a linear fit was applied to the data; the slope of this linear fit gives the sensitivity of the sensor and can either be expressed in EOT shift per RIU [nm/RIU] or in relative EOT shift per RIU [%/RIU]. In addition, the theoretical limit of detection can be calculated as LoD = 3σ_N_/S.

#### 2.2.2. Optical Detection of Bacterial Lysate

As the application of interest for this study is the bacterial detection, PSi membranes passivated with Al_2_O_3_ were tested out for the detection of *Bacillus cereus* lysate. The protocol of detection is previously described [21]. In short, a bacterial suspension of *B. cereus* was incubated for 30 min at 30 °C with the PlyB221 endolysin, encoded by the Deep-Blue phage targeting *B. cereus* [26]. During this incubation, the endolysins selectively lysed the bacteria. The bacterial lysate suspension was then flown through the PSi membranes, which was optically monitored using the setup detailed in Section 2.1.3. Optical spectra were recorded every 10 s and translated into relative EOT changes, as described above. For the oxide coated membranes, the flow was, however, considerably lowered (0.5 to 1 µL/min) compared to the results presented in [21] because of the reduction of pore size and the different wettability of the porous matrix. Moreover, the concentration of lytic agent (PlyB221) was halved, and glycerol was added for a better conservation of the endolysin.

## 3. Results and Discussion

### 3.1. PSi Characterization

#### 3.1.1. Porosity, Thickness and Pore Morphology

After their fabrication process and passivation (either low temperature oxidation or low temperature oxidation and ALD), the membranes were characterized by SEM and SLIM, in order to determine their thickness, porosity, optical properties and pore morphology. The porosity, thickness and skeleton refractive index (n_skeleton_) computed from the SLIM method are detailed in Table 2. Although the quantity of oxide deposited on the passivated samples is unknow, the porosity and n_skeleton_ could be approximated under the hypothesis that the layer thickness did not significantly change after ALD. As can be observed in Table 2, slight variations are still possible, but these can also be attributed to the fact that the studied samples were fabricated in different batches and slight variations in the wafer resistivity resulted in different porous silicon properties. The thickness measurements were also corroborated by cross-sectional views of the membranes, in which an average thickness of 3.60 ± 0.35 µm was observed for the first porous layer. Top view images on SEM (Figure S2) enabled the determination of the average pore size.

The data presented in Table 2 shows a large decrease in porosity after ALD as well as a reduction on average pore size of the sensing layer. This confirms the actual deposition of metal oxide materials inside the porous matrix. Moreover, the significant decrease in open porosity can be attributed to the clogging of the top part of the smaller pores during the ALD. The refractive index is also impacted by the deposition. Furthermore, an increase in optical signal intensity was observed (Figure S3), which was attributed to the higher relative permittivity (or dielectric constants) of the metal oxides (9 for Al_2_O_3_, 80 for TiO_2_ and 25 for HfO_2_, approximate values) compared to SiO_2_ (3.9) [27].

#### 3.1.2. Porous Structure Passivation

**Figure 3:**
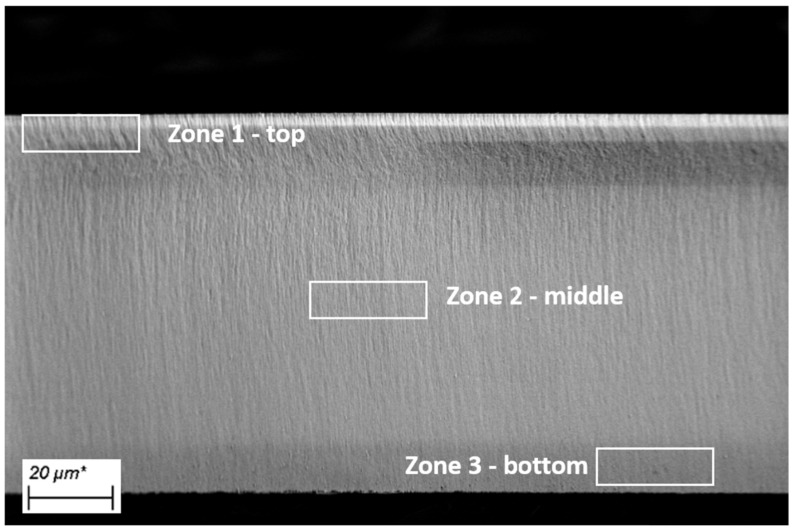
Scanning electron microscopy (SEM) cross-section image of a multi-layered porous silicon membranes, showing the zones defined for Energy Dispersive X-ray Spectroscopy (EDX).

The chemical composition of the metal oxide depositions was first characterized by EDX. Three zones were first evaluated in order to assess the uniformity of the coatings: top, middle and bottom, as depicted in Figure 3.

**Figure 4:**
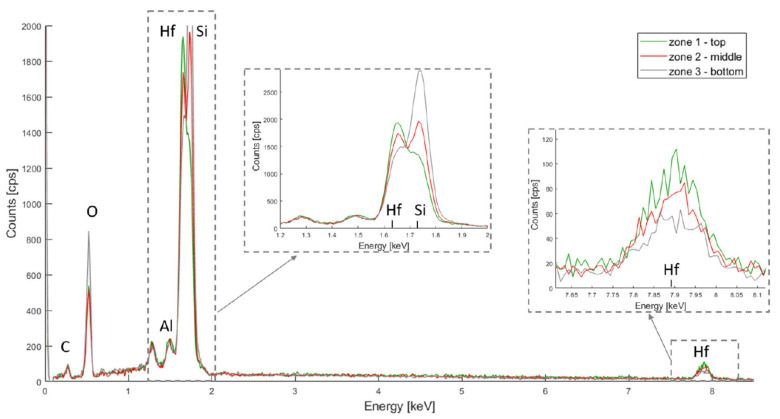
PSiO_2_/HfO_2_ EDX spectra for the top, middle and bottom of the porous membrane.

**Figure 5:**
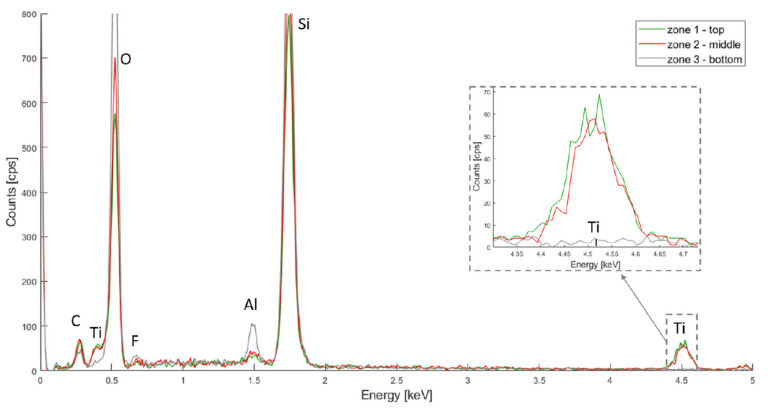
PSiO_2_/TiO_2_ EDX spectra for the top, middle and bottom of the porous membrane.

**Figure 6:**
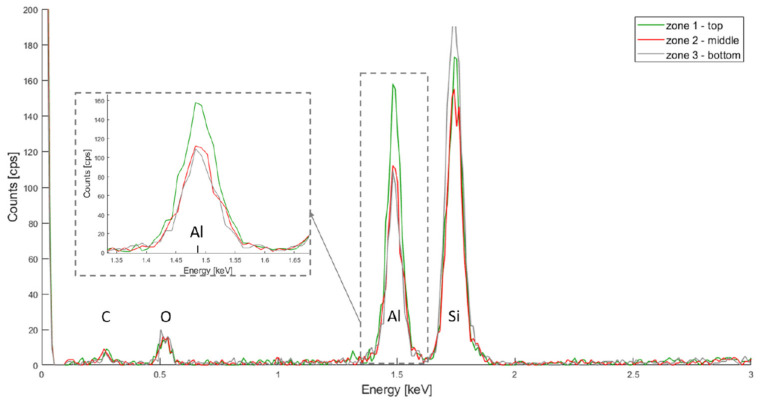
PSiO_2_/Al_2_O_3_ EDX spectra for the top, middle and bottom of the porous membrane.

The resulting spectra for HfO_2_, TiO_2_ and Al_2_O_3_ are depicted in Figure 4, Figure 5 and Figure 6 respectively. In all three spectra, the peak corresponding to the Kα band of oxygen, at 0.525 keV, can be easily observed. Some aluminium contamination at the Kα band, 1.486 keV, is also visible and can be linked to the aluminium backside hard mask used for DRIE.

In Figure 4, hafnium is represented by two peaks at 7.898 keV and 1.644 keV (for the Lα and M bands respectively) and the silicon Kα band is at 1.739 keV. The Hf M band is located very close to the Si Kα band, and the peak overlap. The Si Kα band is, however, more dislocated for the Hf M band for the bottom layer, while the opposite can be observed for the top layer. This suggests that, although HfO_2_ covers the entire membrane depth, more oxide has accumulated on the top part.

In Figure 5, the titanium is also represented by two peaks 4.508 keV (Kα) and 0.452 keV (Lα), and the oxygen Kα band is at 0.525 keV. An overlap can be observed between the Ti Lα band and the O Kα band. When amplifying the Ti Lα peak, signals of similar intensity can be seen for the top and middle layer, but no signal is measured for the bottom. This might suggest an uneven coating, as that the precursors may not penetrate deeply enough into the PSi layer.

Finally, in Figure 6, The Kα bands for silicon, oxygen and aluminium are located at 1.739 keV, 0.525 keV and 1.486 keV, respectively. The aluminium signal is higher for the top layer, but also present in the middle and bottom layers. This same hypothesis as for the HfO_2_ deposition can be made: more oxide was deposited on the top layer.

For each type of metal oxide, line scans were also performed (Figure S4), tracking the presence of metals through the cross section of the membranes. From these measurements, it can be observed that all depositions were uniform, except for the bottom of the PSiO_2_/TiO_2_ membranes. It was, however, observed that this was due to an artifact linked to the cutting of the sample, as the cross-section was not entirely flat.

Compared to studies performed on PSi layers [19,25,28], the membrane morphology affords a better diffusion of the precursors inside the porous matrix and therefore a more uniform in-depth coating. A depth effect is still noticeable, but of much less extent considering the thickness of the PSi membranes.

In order to determine the chemical composition of the coatings inside the membrane, XPS measurements were performed. Both the front- and the back side of the membranes were analysed. The full spectra for each type of ALD are available in Figures S5–S7.

**Figure 7:**
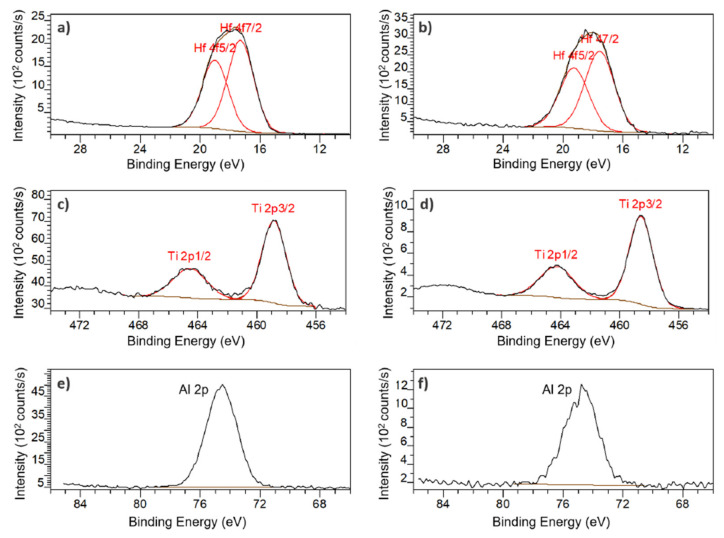
High resolution XPS core level spectra: Hf 4f on PSiO_2_/HfO_2_ membrane (**a**) front, and (**b**) back; Ti 2p on PSiO_2_/TiO_2_ membrane (**c**) front and (**d**) back; Al 2p on PSiO_2_/Al_2_O_3_ membrane (**e**) front and (**f**) back.

The XPS survey spectra PSiO_2_/HfO_2_ presented mainly Hf, O and C but also Si, F and P. The presence of fluorine, especially at the backside, can be explained by contamination due to the SF_6_ gas used during the DRIE step but the origin of the phosphorous signal remains unknown. Figure 7a,b show the Hf 4f spectra for the front and the back of the membrane. After curve fitting, two peaks are observed at 17 eV and 19 eV on both the front- and back side of the membrane, attributed to the binding energies of Hf 4f_7/2_ and Hf 4f_5/2_, respectively. These peaks indicate a valence state of +4 for hafnium and validate that the product of the ALD process is indeed HfO_2_.

For the PSiO_2_/TiO_2_, peaks for Ti, O and C but also Si, and F can be observed in the XPS survey spectra. The presence of fluorine can once again be attributed to the DRIE step. Figure 7c,d reveal two peaks at 459 eV and 464 eV, both on front and back, which correspond to Ti 2p_3/2_ and Ti 2p_1/2_, respectively. Theses peaks also indicating a valence state of +4 for titanium and corroborate the deposition of TiO_2_.

In the XPS survey spectra of PSiO_2_/Al_2_O_3_, the presence of mainly Al, O and C but also F contamination can be observed. In Figure 7e,f, a peak at 74.5 eV can be observed on both front and back. This peak corresponds to the Al 2p binding energy and indicated a +3 valence, validated that Al_2_O_3_ is indeed the deposited product.

### 3.2. Stability in Aqueous Media

**Figure 8:**
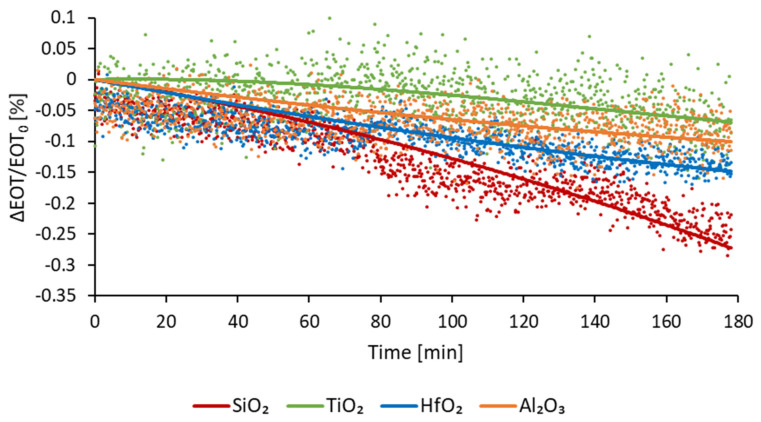
Relative EOT optical response of passivated membranes as a function of time while exposed to a continuous PBS flow.

The optical stability of the coated membranes was assessed in a flow of PBS. This buffer was chosen because of its physiological pH. The stability, expressed in relative EOT change over time ((EOT − EOT_0_)/EOT_0_ %), is measured for 180 min. Figure 8 depicts the change of relative EOT overtime for all three type of ALD coatings, compared to PSiO_2_ membranes. Each curve represents the average data measured for at least five samples.

For PSiO_2_ membranes an increase in relative EOT is observed, of −0.24%. This decrease is consistent with the dissolution of oxidative degradation of the PSi reported in the literature [16,25]. For the PSiO_2_/TiO_2_ membranes, a slight deviation of −0.06% is observed after 3 h, therefore exhibiting an improved stability. During the PSiO_2_/Al_2_O_3_ stability testing, a slow decrease in relative EOT was observed, reaching −0.09% after 3 h. For PSiO_2_/HfO_2_ membranes, a gradual decrease in relative EOT was also observed, reaching −0.14% after 3 h.

These results indicate that coating of metal oxides via ALD does indeed improve the stability of porous silicon. Based on the presented results, the TiO_2_ coating can be qualified as the most stable one. However, besides stability, another important factor that must be considered, especially for optical application, is the noise level of the optical measurement, as well as the sensitivity of the sensing. Indeed, lower noise levels and a higher sensitivity enable lower limits of detection. The calculated noise levels, sensitivity and LoD for each type of membrane are detailed in Table 3.

From this analysis, it can be observed that although the TiO_2_ coating is the most stable one, it is also the one with the highest noise level. For PSiO_2_/HfO_2_ and PSiO_2_/Al_2_O_3_ membranes, the noise level is much lower and the LoD is reduced by nearly a factor of five and a factor of four, respectively. Moreover, the sensitivity of the TiO_2_ coated membranes is the lowest of all four types of sensors. For optical applications, the coating that shows the best compromise between stability overtime, sensitivity and reduced noise level is therefore Al_2_O_3_.

### 3.3. Preliminary Results for the Optical Detection of Bacterial Lysate

**Figure 9:**
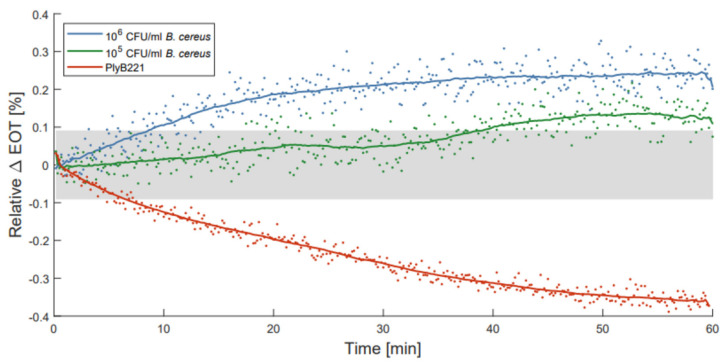
Relative effective optical thickness (EOT) shift measured on a PSi membrane for 1 h in in PlyB221 endolysin suspension (red), and in a *B. cereus* lysate at 10^6^ CFU/mL (blue) and at 10^5^ CFU/mL (green) concentrations.

To illustrate the use of ALD passivated membranes, optical detections of *B. cereus* bacterial lysate were performed as described by Vercauteren et al. [21]. PSiO_2_/Al_2_O_3_ membranes were selected for these measurements, as they exhibit the best compromise between stability and noise level. Before filtering the bacterial lysate through each membrane, PBS was flown through the detection setup for 10 to 15 min to make sure the membranes were fully wet. This reduced transitional period was observed to be sufficient to fully wet the PSiO_2_/Al_2_O_3_ membranes in previous measurements. The changes in relative EOT were measured over 1 h for two initial concentrations of *B. cereus*, namely 10^6^ CFU/mL and 10^5^ CFU/mL. These measurements were compared to a control test, where only the PlyB221 endolysin was present in the suspension. Figure 9 illustrates the change in relative EOT overtime for all three types of measurements, with the grey area indicating the theoretical limit of detection of the PSiO_2_/Al_2_O_3_ membranes. The control test with PlyB221 exhibits a sharp decrease in relative EOT, reaching −0.36% which was unexpected when compared to the results presented in [21]. This seems to indicate an accelerated degradation of the porous matrix, and might be linked to the addition of glycerol to the endolysin suspension. Further investigations are needed to fully understand the source of this degradation.

For the bacterial lysate detection, the measured EOT shifts are significantly lower than the ones previously presented [21], but this may be linked to two factors: (1) the passivated membranes are less porous because of the presence of the oxide coating on their pore walls, and therefore less sensitive to changes in their environment; and (2) because of a decrease in pore size, the flow speed is also considerably reduced (30 to 40 times lower), hence less lysate is filtered through the membrane. However, the noise level of the passivated membrane is much lower as well, allowing, even with lower EOT changes, the detection of 10^6^ CFU/mL and 10^5^ CFU/mL of *B. cereus* lysate in less than 10 min and 40 min respectively.

**Figure 10:**
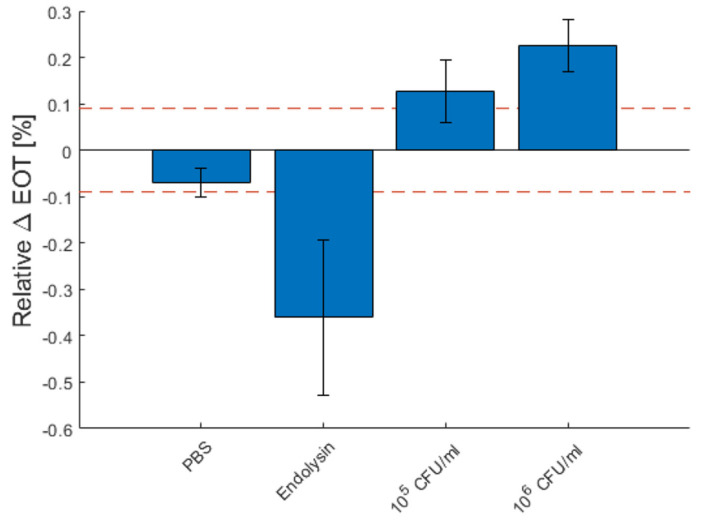
Characteristic relative effective optical thickness (EOT) shift measured on a PSi membrane after 1 h in PBS, in a PlyB221 endolysin suspension and in different concentrations of *B. cereus* lysate. The dashed red line represents the 3σ noise level of the signal measured in PBS. The detection limit is 10^5^ CFU/mL of *B. cereus*.

Figure 10 depicts the total relative EOT changes for each measurement type after 1 h, as well as the standard deviation between each measurement. The red dashed line represents the theoretical limit of detection. The detection of 10^6^ CFU/mL of *B. cereus* lysate was fully reproducible, as all detection was above the dashed line. For 10^5^ CFU/mL, the detection was less reliable, as some measurements were slightly below the detection limit.

While these results show promise in terms of response time (less than 2 h for the entire detection protocol), improvements still must be made to reach the typical limit of detection of PSi biosensors, namely 10 to 10^3^ CFU/mL [29,30,31,32].

## 4. Conclusions and Perspectives

In this work, the conformal deposition of metal oxides on porous silicon membranes was successfully demonstrated. Furthermore, homogeneous distribution of the passivation layer from top to bottom was obtained through low temperature deposition without inducing cracking or collapsing of the membranes. However, ALD passivation resulted in a significant decrease in porosity due to the clogging of small pores.

The passivated samples exhibited improved stability when compared to the low temperature oxidized ones while resulting in a significant reduction of noise for HfO_2_ and Al2O_3_ coatings. A reduction of the limit of detection by a factor of five and four for PSiO_2_/HfO_2_ and PSiO_2_/Al_2_O_3_ passivation, respectively, was obtained.

Finally, the detection of *B. cereus* lysate using a PSiO_2_/Al_2_O_3_ passivated membrane was demonstrated, allowing the detection of 10^5^ CFU/mL concentration in less than one hour. This result confirmed that, even if the flow speed and sensitivity are compromised, the average pore diameter is still large enough for the penetration of the target analytes.

Nevertheless, while the proposed method results in more stable membranes in aqueous media, further adjustments of fabrication parameters are needed to compensate the reduced porosity in order to translate the stability improvements into a lower limit of detection for the biosensor in terms of bacteria concentration.

In terms of perspectives, the ALD temperature could be further increased to obtain denser and better-quality films, up to 150–200 °C [33,34], but the effect on mechanical stability needs to be taken into consideration. The porosity of the layers before metal oxide deposition could also be adjusted to compensate for the decrease that follows the coating, However, this adjustment in fabrication parameters should be handled with care, indeed Increasing too much the porosity and pore size leads to thinner pore walls which can become very fragile, making the whole structure mechanically unstable.

Another passivation technique that does not compromise so much the porosity as ALD could be adopted, such as “hydrocarbonization” [17]. This strategy could replace the oxidation step and even act as a stop layer for the deep reactive ion etching, eliminating one process step.

## Figures and Tables

**Table 1:** Parameters for ALD of different metal oxides in a Fiji F200 ALD reactor.

	HfO_2_	Al_2_O_3_	TiO_2_
Precursor Pulse 1	0.3 s	0.5 s	0.2 s
Wait	120 s	120 s	120 s
Purge	360 s	360 s	360 s
Precursor Pulse 2	0.1 s	0.1 s	0.2 s
Wait	120 s	120 s	120 s
Purge	360 s	360 s	360 s
Total number of cycles	20	40	50
Deposition temperature	100 °C	100 °C	150 °C

**Table 2:** Thickness, open porosity and n_skeleton_ of the sensing layer of the passivated PSi membranes, evaluated by the SLIM method. Pore size was evaluated through SEM image analysis.

	SLIM			SEM
	Thickness [µm]	Open Porosity [%]	n_skeleton_ [RIU]	Pore Size [nm]
PSiO_2_	3.66 ± 0.35	74.7 ± 5.8	2.52 ± 0.17	30.3 ± 14.8
PSiO_2_/HfO_2_	3.67 ± 0.21	56.9 ± 4.5	2.38 ± 0.20	26.4 ± 14.0
PSiO_2_/TiO_2_	3.60 ± 0.13	62.1 ± 6.1	2.57 ± 0.25	25.5 ± 11.4
PSiO_2_/Al_2_O_3_	3.56 ± 0.21	62.4 ± 4.7	2.09 ± 0.17	23.2 ± 12.0

**Table 3:** Noise level, sensitivity and theoretical limit of detection for the passivated PSi membranes.

	σ_N_ [%]	Sensitivity [%/RIU]	LoD ×10^−3^ [RIU]
PSiO_2_	0.080	59.6	4.0
PSiO_2_/HfO_2_	0.019	38.7	1.5
PSiO_2_/TiO_2_	0.089	35.6	7.5
PSiO_2_/Al_2_O_3_	0.030	48.6	1.9

## Supplementary Materials

The following are available online at https://www.mdpi.com/article/10.3390/mi13010010/s1, Figure S1: Schematic representation of the noise level analysis procedure: (1) the data is fit using a moving average (low pass filter with coefficient equal to the reciprocal of the span, span = 0.15), (2) the noise is calculated by taking the difference between the data and the fit, (3) a normal probability distribution is plotted from the calculated noise, (4) a Gaussian fit is applied to the distribution, allowing to extract the noise level σ_N_; Figure S2: SEM images of the surface of porous silicon membranes: PSiO_2_, PSiO_2_/HfO_2_, PSiO_2_/Al_2_O_3_ and PSiO_2_/TiO_2_; Figure S3: Interferometric spectra obtained for (a) PSiO_2_/HfO_2_, (b) PSiO_2_/TiO_2_ and (c) PSiO_2_/Al_2_O_3_, before and after the ALD, at same integration time (t = 1 s); Figure S4: EDX line scan showing deposition on the full depth of the membrane: (a) PSiO_2_/HfO_2_, (b) PSiO_2_/TiO_2_ and (c) PSiO_2_/Al_2_O_3_. The red dotted lines indicate the approximate location of the interfaces between first/second and second/third layers; Figure S5: XPS survey spectra of the PSiO_2_/HfO_2_ membrane front (black) and back (red). The main core levels are labeled. The data are normalized to each C-(C,H) component of the C 1s peak and separated vertically; Figure S6: XPS survey spectra of the PSiO_2_/TiO_2_ membrane front (black) and back (red). The main core levels are labeled. The data are normalized to each C-(C,H) component of the C 1s peak and separated vertically; Figure S7: XPS survey spectra of the PSiO_2_/Al_2_O_3_ membrane front (black) and back (red). The main core levels are labeled. The data are normalized to each C-(C,H) component of the C 1s peak and separated vertically.

## References

[1] Tieu T., Alba M., Elnathan R., Cifuentes-Rius A., Voelcker N.H. (2019). Advances in Porous Silicon–Based Nanomaterials for Diagnostic and Therapeutic Applications. Adv. Ther..

[2] Jung Y., Huh Y., Kim D. (2021). Recent Advances in Surface Engineering of Porous Silicon Nanomaterials for Biomedical Applications. Microporous Mesoporous Mater..

[3] Abu-Thabit N., Ratemi E. (2020). Hybrid Porous Silicon Biosensors Using Plasmonic and Fluorescent Nanomaterials: A Mini Review. Front. Chem..

[4] Moretta R., De Stefano L., Terracciano M., Rea I. (2021). Porous Silicon Optical Devices: Recent Advances in Biosensing Applications. Sensors.

[5] Canham L. (2014). Handbook of Porous Silicon.

[6] Herino R., Bomchil G., Barla K., Bertrand C., Ginoux J.L. (1987). Porosity and Pore Size Distributions of Porous Silicon Layers. J. Electrochem. Soc..

[7] Harraz F.A. (2014). Porous Silicon Chemical Sensors and Biosensors: A Review. Sens. Actuators B Chem..

[8] Vercauteren R., Scheen G., Raskin J.-P., Francis L.A. (2020). Porous Silicon Membranes and Their Applications: Recent Advances. Sens. Actuators A Phys..

[9] Anderson S.H.C., Elliott H., Wallis D.J., Canham L.T., Powell J.J. (2003). Dissolution of Different Forms of Partially Porous Silicon Wafers under Simulated Physiological Conditions. Phys. Status Solidi A Appl. Res..

[10] De Stefano L. (2019). Porous Silicon Optical Biosensors: Still a Promise or a Failure?. Sensors.

[11] Sailor M.J. (2012). Porous Silicon in Practice: Preparation, Characterization and Applications.

[12] Pap A.E., Kordás K., Tóth G., Levoska J., Uusimäki A., Vähäkangas J., Leppävuori S., George T.F. (2005). Thermal Oxidation of Porous Silicon: Study on Structure. Appl. Phys. Lett..

[13] Stewart M.P., Robins E.G., Geders T.W., Allen M.J., Choi H.C., Buriak J.M. (2000). Three Methods for Stabilization and Functionalization of Porous Silicon Surfaces via Hydrosilylation and Electrografting Reactions. Phys. Status Solidi A Appl. Res..

[14] Terracciano M., De Stefano L., Rendina I., Oliviero G., Piccialli G., Borbone N., Rea I. Aminosilane-Modified Mesoporous Oxidized Silicon for in Situ Oligonucleotides Synthesis and Detection. Proceedings of the 2014 Fotonica AEIT Italian Conference on Photonics Technologies.

[15] Terracciano M., Rea I., Politi J., De Stefano L. (2013). Optical Characterization of Aminosilane-Modified Silicon Dioxide Surface for Biosensing. J. Eur. Opt. Soc. Rapid Publ..

[16] Shabir Q., Webb K., Nadarassan D.K., Loni A., Canham L.T., Terracciano M., Stefano L.D., Rea I. (2018). Quantification and Reduction of the Residual Chemical Reactivity of Passivated Biodegradable Porous Silicon for Drug Delivery Applications. Silicon.

[17] Salonen J., Björkqvist M., Laine E., Niinistö L. (2004). Stabilization of Porous Silicon Surface by Thermal Decomposition of Acetylene. Appl. Surf. Sci..

[18] Franssila S. (2004). Introduction to Microfabrication.

[19] Iatsunskyi I., Kempiński M., Jancelewicz M., Załęski K., Jurga S., Smyntyna V. (2015). Structural and XPS Characterization of ALD Al_2_O_3_ Coated Porous Silicon. Vacuum.

[20] Rasson J., Francis L.A. (2018). Improved Stability of Porous Silicon in Aqueous Media via Atomic Layer Deposition of Oxides. J. Phys. Chem. C.

[21] Vercauteren R., Leprince A., Mahillon J., Francis L.A. (2021). Porous Silicon Biosensor for the Detection of Bacteria through Their Lysate. Biosensors.

[22] He Y., Leïchlé T. (2017). Fabrication of Lateral Porous Silicon Membranes for Planar Microfluidics by Means of Ion Implantation. Sens. Actuators B Chem..

[23] He Y., Bourrier D., Imbernon E., Leïchlé T. Lateral Porous Silicon Membranes with Size and Charge Selectivity. Proceedings of the 2017 IEEE 12th International Conference on Nano/Micro Engineered and Molecular Systems (NEMS).

[24] Gautier G., Kouassi S. (2015). Integration of Porous Silicon in Microfuel Cells: A Review. Int. J. Energy Res..

[25] Rasson J. (2018). Stable Porous Silicon-Based Optical Sensing Platform towards Detection of Bacterial Lysis. Ph.D. Thesis.

[26] Leprince A., Nuytten M., Gillis A., Mahillon J. (2020). Characterization of PlyB221 and PlyP32, Two Novel Endolysins Encoded by Phages Preying on the Bacillus Cereus Group. Viruses.

[27] Robertson J. (2004). High Dielectric Constant Oxides. Eur. Phys. J. Appl. Phys..

[28] Iatsunskyi I., Jancelewicz M., Nowaczyk G., Kempiński M., Peplińska B., Jarek M., Załęski K., Jurga S., Smyntyna V. (2015). Atomic Layer Deposition TiO_2_ Coated Porous Silicon Surface: Structural Characterization and Morphological Features. Thin Solid Films.

[29] Tenenbaum E., Segal E. (2015). Optical Biosensors for Bacteria Detection by a Peptidomimetic Antimicrobial Compound. Analyst.

[30] Yaghoubi M., Rahimi F., Negahdari B., Rezayan A.H., Shafiekhani A. (2020). A Lectin-Coupled Porous Silicon-Based Biosensor: Label-Free Optical Detection of Bacteria in a Real-Time Mode. Sci. Rep..

[31] Urmann K., Walter J.-G., Scheper T., Segal E. (2015). Label-Free Optical Biosensors Based on Aptamer-Functionalized Porous Silicon Scaffolds. Anal. Chem..

[32] Tang Y., Li Z., Luo Q., Liu J., Wu J. (2016). Bacteria Detection Based on Its Blockage Effect on Silicon Nanopore Array. Biosens. Bioelectron..

[33] Groner M.D., Fabreguette F.H., Elam J.W., George S.M. (2004). Low-Temperature Al_2_O_3_ Atomic Layer Deposition. Chem. Mater..

[34] Porro S., Jasmin A., Bejtka K., Conti D., Perrone D., Guastella S., Pirri C.F., Chiolerio A., Ricciardi C. (2016). Low-Temperature Atomic Layer Deposition of TiO_2_ Thin Layers for the Processing of Memristive Devices. J. Vac. Sci. Technol. A Vac. Surf. Films.

