# Supplementary material for: Passivated Porous Silicon Membranes and Their Application to Optical Biosensing
Source: Micromachines (Basel). 2021 Dec 22;13(1):10. doi: 10.3390/mi13010010 (PMC8779296; doi:10.3390/mi13010010)
Supplement: Supplementary file 1 [file micromachines-13-00010-s001.zip › micromachines-1507867-supplementary.pdf]

Passivated Porous Silicon Membranes and their Application to Optical Biosensing

Clara Whyte Ferreira [1], Roselien Vercauteren [1,*] and Laurent A. Francis [1]

[1] Institute of Information and Communication Technologies Electronics and Applied Mathematics, UCLouvain, 1348 Louvain-la-Neuve, Belgium

* Correspondence: roselien.vercauteren@uclouvain.be;

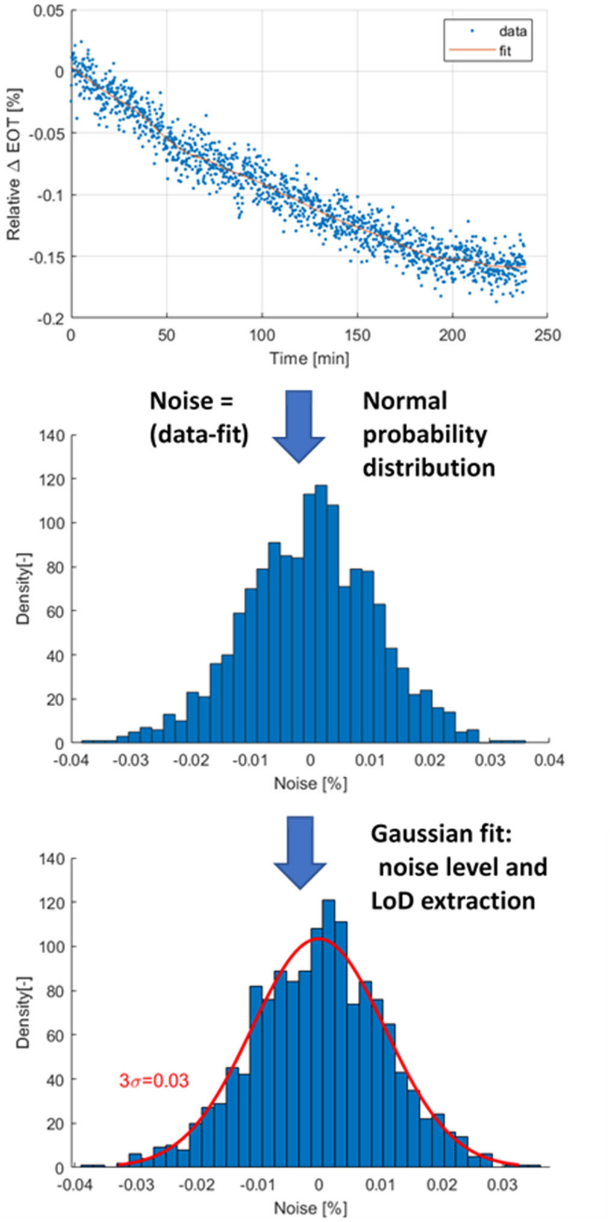

Figure S1. Schematic representation of the noise level analysis procedure: (1) the data is fit using a moving average (low pass filter with coefficient equal to the reciprocal of the span, span=0.15), (2) the noise is calculated by taking the difference between the data and the fit, (3) a normal probability distribution is plotted from the calculated noise, (4) a Gaussian fit is applied to the distribution, allowing to extract the noise level σ_N.

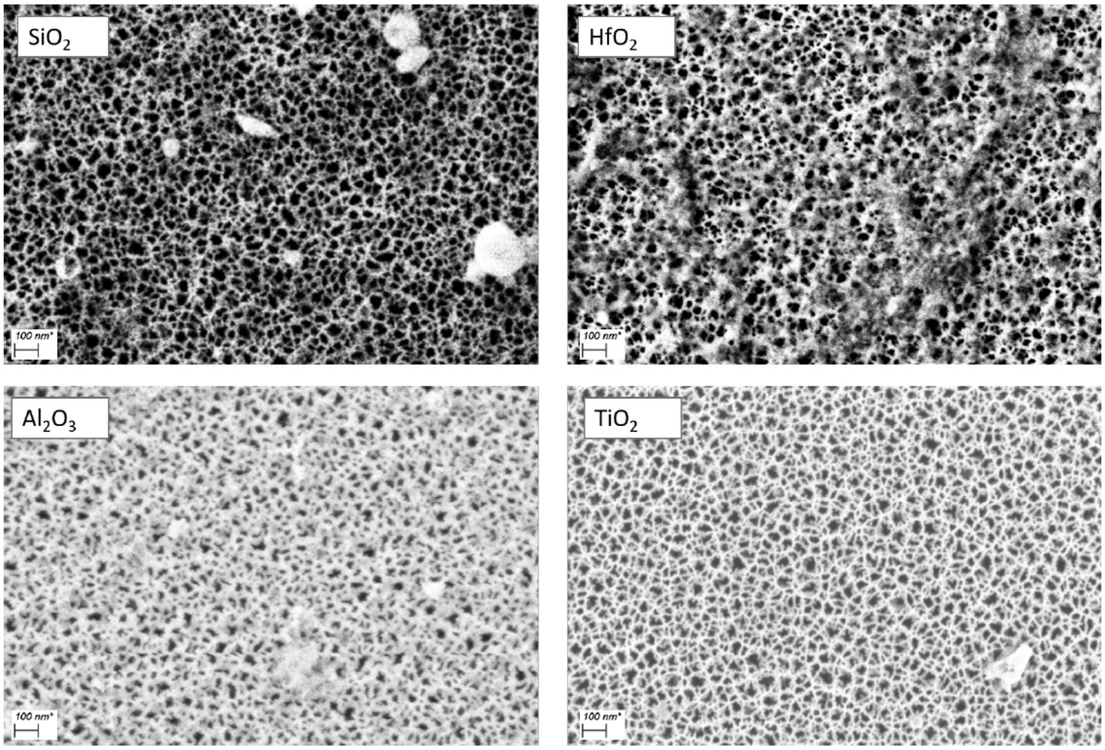

Figure S2. SEM images of the surface of porous silicon membranes: PSiO$_2$, PSiO$_2$/HfO$_2$, PSiO$_2$/Al$_2$O$_3$ and PSiO$_2$/TiO$_2$.

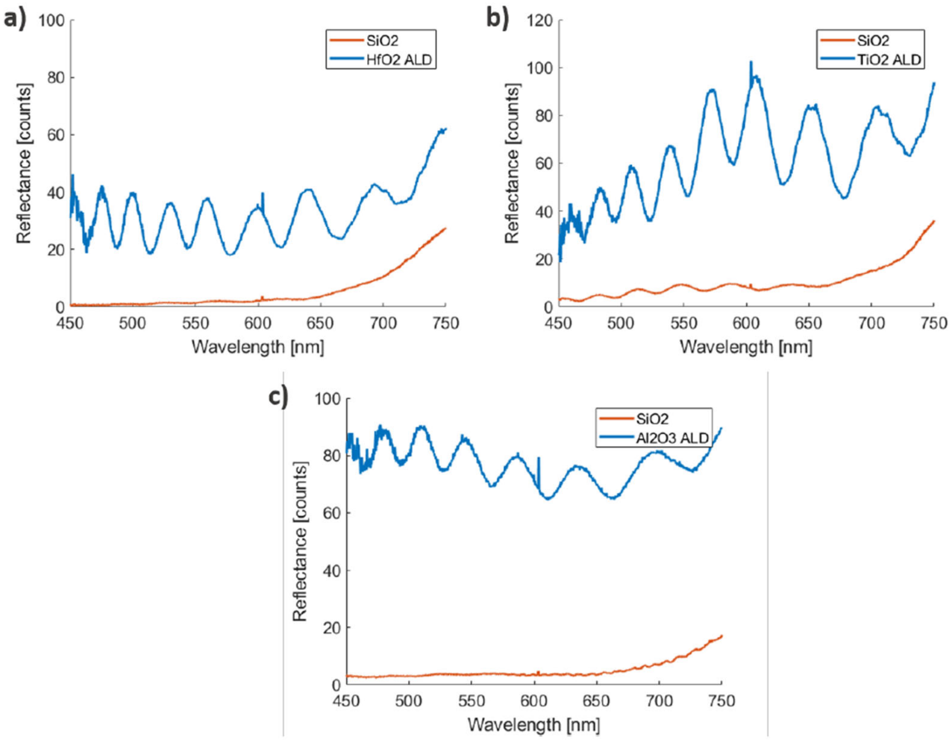

Figure S3. Interferometric spectra obtained for (a) PSiO$_2$/HfO$_2$, (b) PSiO$_2$/TiO$_2$, and (c) PSiO$_2$/Al$_2$O$_3$, before and after the ALD, at same integration time (t = 1s).

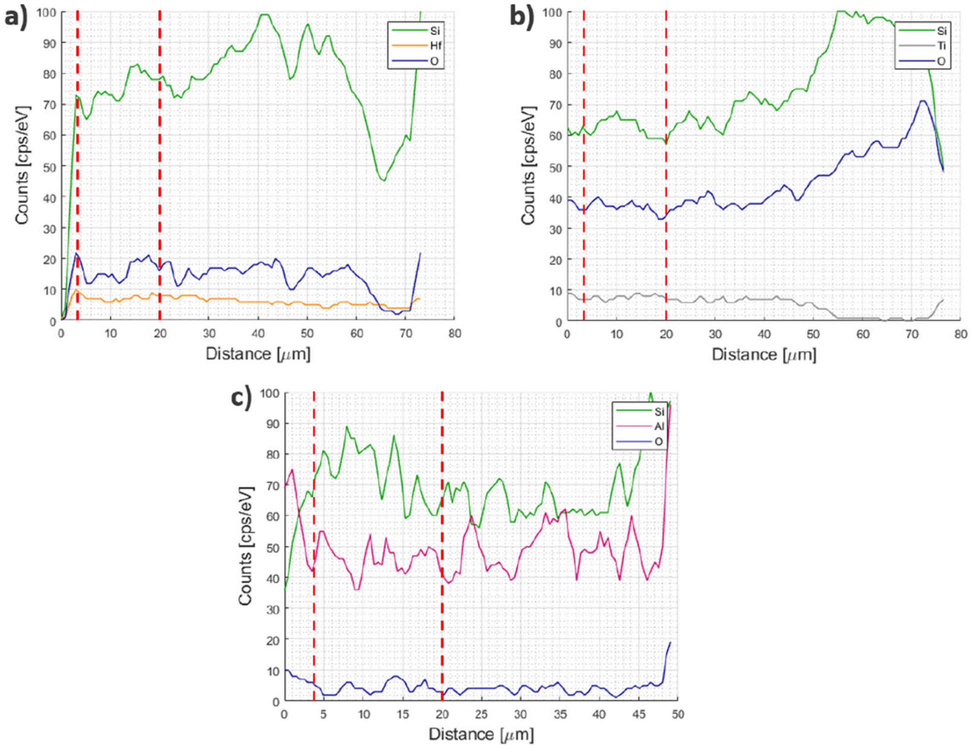

Figure S4. EDX line scan showing deposition on the full depth of the membrane: (a) $PSiO_2/HfO_2$, (b) $PSiO_2/TiO_2$, and (c) $PSiO_2/Al_2O_3$. The red dotted lines indicate the approximate location of the interfaces between first/second and second/third layers.

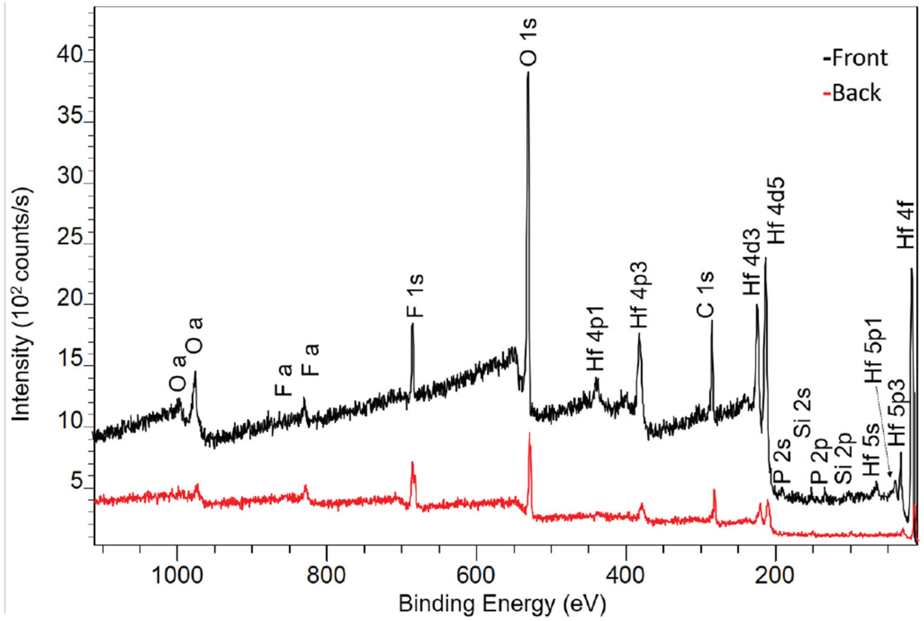

Figure S5. XPS survey spectra of the $PSiO_2/HfO_2$ membrane front (black) and back (red). The main core levels are labeled. The data are normalized to each C-(C,H) component of the C 1s peak and separated vertically.

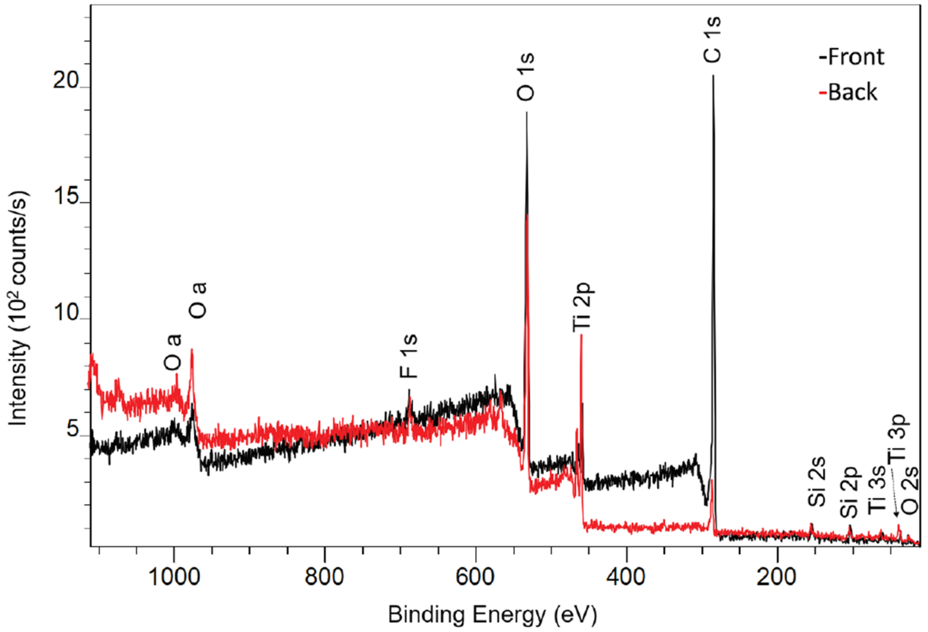

Figure S6. XPS survey spectra of the $PSiO_2/TiO_2$ membrane front (black) and back (red). The main core levels are labeled. The data are normalized to each C-(C,H) component of the C 1s peak and separated vertically.

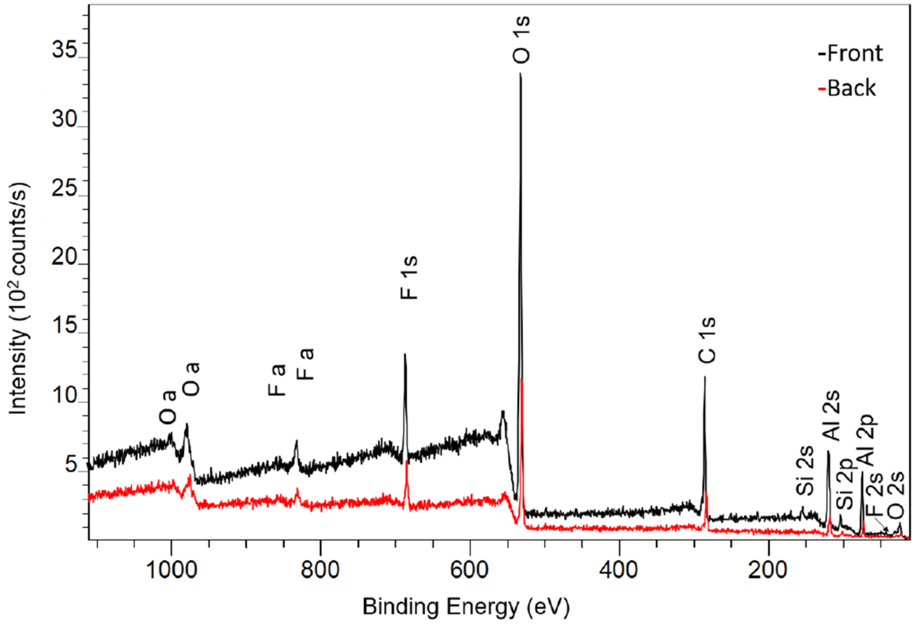

Figure S7. XPS survey spectra of the $PSiO_2/Al_2O_3$ membrane front (black) and back (red). The main core levels are labeled. The data are normalized to each C-(C,H) component of the C 1s peak and separated vertically.
